# VIEWS ON AGING: NEW PERSPECTIVES FOR THEORY AND RESEARCH

**DOI:** 10.1093/geroni/igz038.2894

**Published:** 2019-11-08

**Authors:** Verena Klusmann, Anna E Kornadt

**Affiliations:** 1 University of Konstanz, Konstanz, Germany; 2 Bielefeld University, Bielefeld, Germany

## Abstract

Over the past 20 years, research on views on aging has substantiated their importance for successful development and sustained quality of life over the full length of the life span. However, a deep understanding of the origins of views on aging and the underlying processes of their lifespan development and manifestation is lacking. Since 2017, the scientific network “Images of Aging” funded by the German Research Foundation (http://www.health.uni-konstanz.de/images-of-aging) assembles national and international renowned experts in the field. The network engages in empirical clarifications on both the distinctness and validity of the construct (contribution of Klusmann et al.) as well as in critically reviewing terminology and measures of views on aging (contribution of Notthoff et al.). The network aims to help clarifying the dynamic interplay of determinants and outcomes in the context of health (contribution of Wolff et al.) as well as disentangling intra- and intergenerational stereotypic perceptions (contribution of Kornadt et al.). Both of these are understudied issues with highly practical implications for two of the largest demographic challenges: shaping the coexistence of generations as well as providing adequate health care supply. Integrating both pertinent theoretical approaches and empirical findings the network regards views on aging under a lifespan perspective. Recently, it suggested three core principles of views on aging regarding lifelong bio-psycho-social development, their multidimensional nature, and their impact across life. These considerations provide a background for an integrative discussion of the symposium’s contributions.

